# Developing a Multi-Method Approach for Understanding Cellular Uptake and Biological Response: Investigating Co-Exposure of Macrophage-like Differentiated THP-1 Cells to Al_2_O_3_ and CeO_2_ Nanoparticles

**DOI:** 10.3390/molecules30071647

**Published:** 2025-04-07

**Authors:** Yves Uwe Hachenberger, Benjamin Christoph Krause, Fabian Lukas Kriegel, Philipp Reichardt, Jutta Tentschert, Harald Jungnickel, Frank Stefan Bierkandt, Peter Laux, Ulrich Panne, Andreas Luch

**Affiliations:** 1 Department of Chemical & Product Safety, German Federal Institute for Risk Assessment (BfR), Max-Dohrn-Strasse 8-10, 10589 Berlin, Germany; 2 Federal Institute for Materials Research and Testing (BAM), Richard-Willstätter-Strasse 11, 12489 Berlin, Germany

**Keywords:** nano, co-exposition, viability changes, ICP-MS, ToF-SIMS

## Abstract

The use of different nanoparticles (NPs) is increasing in a wide variety of everyday products. Nevertheless, most studies concerning NP risk assessment have evaluated exposure scenarios involving a single kind of NP. A stepwise study distinguishing between the effects resulting from exposure to one kind of NP and those resulting from different co-exposure scenarios to ${\mathrm{Al}}_{2}\;O_{3}$ and ${\mathrm{CeO}}_{2}$ NPs at concentrations below acute toxicity was conducted with different analytical techniques. As a starting point, WST-1 viability assays were performed to assess whether the chosen exposure concentrations resulted in any acute loss of viability, which would hamper further insight into the cellular response to NP exposure. Then, data on NP dissolution and uptake were obtained via single-particle inductively coupled plasma–mass spectrometry (spICP-MS) and microwave-assisted ICP-MS. Additionally, time-of-flight secondary ion mass spectrometry (ToF-SIMS) was performed to check for differences in the biological response to the exposure scenarios at the single-cell level. It was found that the proposed combined techniques provide insight into changes in biological responses as well as cellular metal contents among the exposure scenarios. In this work, a comprehensive tiered analytical strategy for evaluating the biological responses to challenging exposure scenarios is provided. The results highlight the necessity of selecting situations more closely resembling real life—including concentrations below acute toxicity and potential interactions due to multiple NPs—when estimating potential health risks. These findings thus provide a foundation and an incentive for further research into the complex processes leading to the observed effects.

## 1. Introduction

Due to their broad range of unique properties, nanomaterials (NMs) are being used in an increasing number of everyday products [1]. Thus, exposure to a multitude of different NMs in everyday life is expected [2,3]. The variety of existing and applied NMs and their physicochemical properties, such as size, shape, composition, and redox potential, determine their interactions and fate in different environments [4,5,6]. This plethora of different materials can lead to challenges if a single generalized analytical method is applied to characterize NMs in terms of their properties as well as their toxicity, as certain analytical techniques might be unsuitable [7,8,9,10,11]. This is also the case when it comes to evaluating the actual exposure to NMs and their cellular interactions, making risk assessment challenging [12,13]. The complexity of this situation is further increased when possible interactions between different NMs or NMs and their environment are taken into consideration [14,15,16]. Due to these framework conditions, scientific research has mainly focused on exposure scenarios involving a single kind of NM [17,18]. Nevertheless, different co-exposure scenario experiments have been described, showing the relevance of investigating them. An example of increased toxicity was shown after rainbow trout were simultaneously exposed to two different nanomaterials: CuO nanoparticles (NPs) and ZnO NPs [19]. In a study with carp and different exposure scenarios with ${\mathrm{TiO}}_{2}$ NPs and AgNPs, an increase in Ag NP toxicity was observed. Additionally, NP fate and biological impact were influenced by the respective (co)-exposure scenario [20]. Furthermore, the complexity of the possible interactions of NPs and their subsequent biological impacts is further increased once different kinds of substances are taken into consideration, such as metals, pharmaceutical drugs, or vitamins [21,22,23].

Ceriumdioxide NPs are emitted in the exhaust gases of cars [24]. Studies on ${\mathrm{CeO}}_{2}$ NPs have reported different effects in cell culture experiments, such as increases in oxidative stress and apoptosis, as well as a protective influence, attributed to their antioxidant properties [25,26,27]. These results make ${\mathrm{CeO}}_{2}$ NPs a worthwhile candidate for studying the different connections between NPs and cellular responses [28]. Aluminum is a naturally occurring and ubiquitous element [29,30]. Due to its chemical nature, it is mainly present as aluminum, aluminum oxide, or aluminum hydroxide. Aluminum oxide is used during the manufacturing process of a wide range of everyday products, such as ceramics, electrical insulators, paper, light bulbs, and concrete. It is also used as a food additive [31,32,33]. Several forms of aluminum oxide exist, with the alpha and gamma forms being the major ones. Due to its oxidized surface, the solubility of gamma aluminum oxide is expected to be low. However, depending on the pH, dissolution may occur. Therefore, instead of dissolution outside the cells, ionic aluminum can have cytotoxic effects after uptake and lysosomal digestion. Research suggests that different aluminum species pose health risks [34]. Due to their potential for interaction with ${\mathrm{CeO}}_{2}$ NPs after uptake, gamma-${\mathrm{Al}}_{2}\;O_{3}$ NPs were selected as the second exposure material in our experiments. The joint research centre (JRC) repository material NM-212 and NMs from Iolitec were selected for ${\mathrm{CeO}}_{2}$ and ${\mathrm{Al}}_{2}\;O_{3}$, respectively [35,36,37]. We selected this NM combination for several reasons. As both NMs are used in a wide range of applications, such exposure scenarios are likely to occur in real life [38,39]. Additionally, based on the available research, potential adverse biological and mitigating effects have been reported, indicating this would be an exemplary co-exposure scenario for use in this proof-of-principle study [40,41,42]. Furthermore, both materials have been well described in past work and can be obtained commercially [35,36,37,43,44]. An overview of the key characterization parameters is provided in Table S1. Differentiated THP-1 cells, which have been shown to express macrophage-like behavior, were chosen as a cell culture model [45,46]. An advantage of this model is its potential for investigating effects in the context of different kinds of (co-)exposure scenarios, including potential NP–NP interactions and their effect on cellular responses due to the expected accumulation of NPs.

A commonly used assay to evaluate cell viability changes is the [1]-1 (4-[3-(4-iodophenyl)-2-(4-nitro-phenyl)-2H-5-tetrazolio]-1,3-benzene sulfonate) assay [47]. Here, a photometric evaluation of a biodegradable dye applied to cell culture was used to evaluate overall cellular activity following exposure to certain changes in conditions compared to the unexposed control samples. This widely used technique is fast and robust [48]. However, as no further information is provided during the measurements, the interpretation of the results can be a challenge [49].

A wide range of techniques for characterizing NPs have been developed, evaluated and used based on the properties of interest as well as the sample itself (e.g., a dispersion or a pure powder) [9,50]. For example, the characterization of metallic NPs is possible with single-particle inductively coupled–mass spectrometry (spICP-MS) [51,52]. Complete ionization of the sample solution takes place in plasma. Afterwards, the metallic content can be quantified based on the individual mass-to-charge ratio of the ions. Due to the high time resolution and the high dilution of the samples, the mass of individual particles can be obtained, and their sizes can be calculated based on an assumed spherical shape. To enable the ionization and detection of individual NPs, samples have to be diluted below approximately $10^{6}$ particles $L^{-1}$ in water [53,54]. In this way, the matrix effects based on other sample constituents are reduced, which might influence the results. A system for undiluted injection, optimized for complex sample solutions and called direct injection (DI) spICP-MS, has been described in detail elsewhere [10]. An advantage of this technique is the fast measurement without the need for further sample preparation, which provides insights into particle size distributions based on single-particle information instead of averaged results. Additionally, depending on the baseline level, data about the ionic sample content are obtained. However, complex samples may be challenging to analyze without further sample preparation due to matrix effects. Several digestion methods have been reported in the literature to reduce the burden on the instruments used, such as acid-based microwave or enzymatic digestion [55,56]. A combination of these approaches allows for the determination of the total metal content (microwave digestion (MW) and subsequent ICP-MS-based quantification) as well as the fate of NPs before their interaction with the cell culture model (DI spICP-MS).

Another mass-based analytical technique is time-of-flight–secondary ion mass spectrometry (ToF-SIMS) [57]. Here, an ion beam is focused on a sample spot and ionizes the sample’s top layers. The ions are then analyzed with a time-of-flight analyzer, and the mass spectrum of each three-dimensional point (voxel) is obtained. The obtained data can be used to create 3D images based on selected *m*/*z* values and analyzed with statistical methods to compare the spectral data of different samples. One example is the use of principal component analysis (PCA) to evaluate complex datasets. Differences between sample subsets are identified based on the signal intensity of individual *m*/*z* values in the spectra as well as their contribution to the differences [58,59]. Thus, PCA is a versatile technique for the visualization and characterization of different samples [60,61,62]. However, a solid sample is normally necessary for the high-vacuum conditions of the measurements. Therefore, the sample preparation of biological samples involves a fast-freezing and then a freeze-drying step, which is time-consuming [63]. Another possibility would be measurement at low temperatures after the fast-freezing process [64].

**Figure 1:**
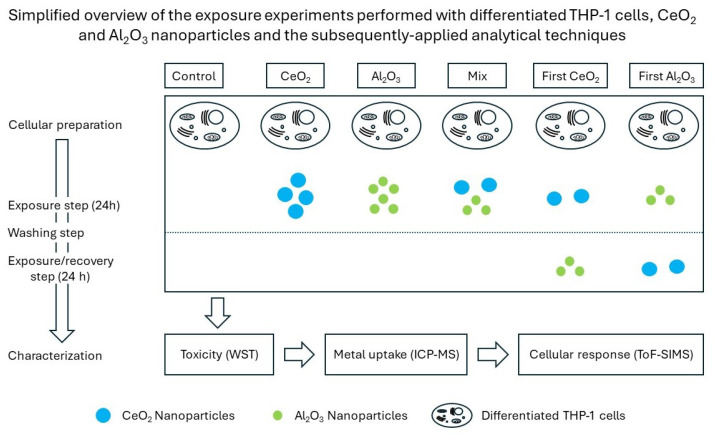
Exposure scheme of differentiated THP-1 cells to NPs and their following characterization.

In this study, the effects of different NP (co-)exposure scenarios with doses below the observed acute toxicity on differentiated THP-1 cells were investigated. The main goal was to evaluate if the fate of the NPs as well as the impact on biological responses could be determined with the techniques applied. The results highlight the importance of using more realistic exposure scenarios due to their influence on cellular responses. The proposed approach can be used as a blueprint to design an analytical workflow that is based on the demands, and results relate to the scientific questions at hand. These questions include the properties of the sample itself, the matrix in which it is contained, the fate of the NPs after their interaction with differentiated THP-1 cells, and their biological impact. In this case, the tiered approach used to evaluate these was as follows: First, an evaluation of viability changes of the exposure scenarios was conducted with a WST-1 assay. If the results were unsuitable, the NP fate after exposure was determined with DI spICP-MS as well as microwave-digestion-based evaluation of the metal content based on the uptake of NPs. If the uptake of NPs is assumed, further verification and insights into the biological response are provided by ToF-SIMS. A summary of this process is provided in Figure 1. In our study, we observed different biological responses in the different exposure scenarios and verified these by combining the results of the aforementioned analytical methods. These insights can be used as a starting point for further investigation into the complex interactions between NPs and their environment as well as into the related biological responses.

## 2. Results and Discussion

### 2.1. Experimental Design

The applied workflow was a stepwise process. With this design, a pragmatic evaluation of various complex exposure scenarios was performed. Therefore, after every experimental part, an evaluation of the results was performed to determine the necessity of further experiments. This was conducted in consideration of the analytical questions to be answered. An overview of the performed exposure experiments and the techniques applied is provided in Figure 1. In this example, the response of differentiated THP-1 cells to different NP exposure scenarios was evaluated. One question of interest concerned the influence of a mixture of these particles or their successive exposure compared to a single dose of one NP.

To evaluate the potential risks, WST-1 assays were first performed to determine the changes in cell viability in different scenarios. As the next step, the dissolution of the NPs in the cell culture media was evaluated to distinguish between the effects of ionic and particulate matter. Due to the macrophage-like behavior, an uptake of the NPs was expected. To gain further insights into the fate of the NPs, the NP uptake based on the assessment of the metal contents was determined via the microwave-assisted digestion of the samples and their characterization with ICP-MS. Finally, the differences in the biological response were analyzed based on the observed variations in the mass spectra of individual cells. The tiered experimental approach provided flexibility in the choice of the experiments based on the individual outcome of the different analytical techniques. If complex analytical interactions are being researched and a broad range of instruments are available, such a strategy is efficient. In this case, the complexity is due to the NPs themselves, their interactions with each other, and their environment, which are influenced by the exposure scenario and the selected biological system. Table S1 provides an overview of the key parameters of the different NPs. Based on these values, different biological responses were expected, which would demonstrate the complexity of the chosen polydisperse NPs. However, real-life scenarios have an even higher degree of complexity, so the limits of the analytical possibilities are pushed even further due to the increased challenge.

### 2.2. Viability Changes

**Figure 2:**
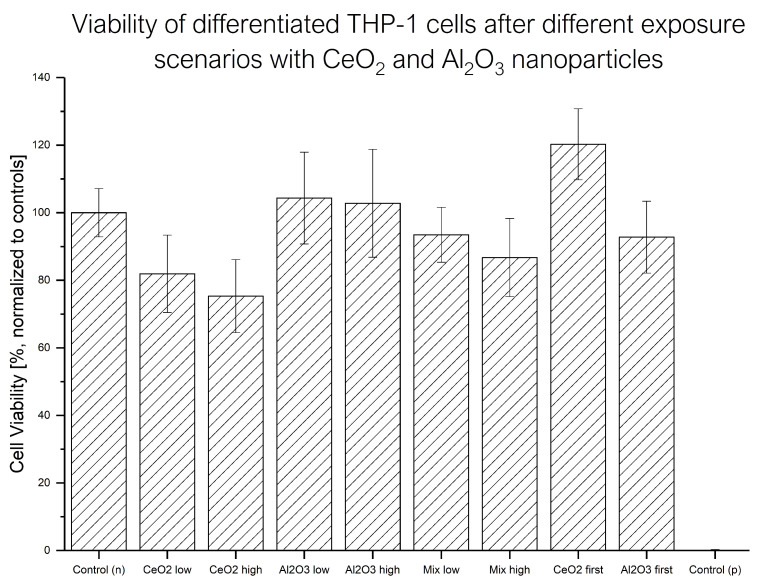
Results of WST-1 assays of differentiated THP-1 cells in different NP exposure scenarios with different NP concentrations (${\mathrm{CeO}}_{2}$ low: 6 g $L^{-1}$, ${\mathrm{CeO}}_{2}$ high: 12 g $L^{-1}$, ${\mathrm{Al}}_{2}\;O_{3}$ low: 6 mg $L^{-1}$, ${\mathrm{Al}}_{2}\;O_{3}$ high: 12 mg $L^{-1}$) from 3 biological replicates.

To determine the relevance of a certain exposure scenario, changes in cell viability provide valuable information. The rapid measurements and straightforward data evaluation make this technique a suitable screening method. An overview of the averaged WST-1 results is shown in Figure 2. The observed viability changes were quite low, and their uncertainty was quite high.

Student’s *t*-tests were performed between each sample group to obtain a measure of the differentiation between the observed viabilities. An overview is provided in Table 1.

The exposure of differentiated THP-1 cells to ${\mathrm{CeO}}_{2}$ resulted in a slight decrease in viability. The effects of ${\mathrm{Al}}_{2}\;O_{3}$ exposure scenarios could not be distinguished from the negative control, and therefore no toxic response was found for either concentration. The exposure scenarios with a mixture of both NPs produced a decrease in cell viability as response, but, due to the uncertainties of the results, the difference was not significant. For all single-exposure experiments (only one NP or a mixture), no significant difference between the different exposure concentrations of NPs was found. However, when the low-concentration exposure to ${\mathrm{CeO}}_{2}$ NP was followed by ${\mathrm{Al}}_{2}\;O_{3}$ NP exposure, increased cell viability was observed compared to the control. When the exposure order of the NPs was reversed, this effect was not observed.

To evaluate the influence of the dissolved NPs, DI spICP-MS measurements were performed in the different exposure scenarios (blank measurements, each kind of NP, and mixture of both NPs) in the used cell culture media with concentrations suitable for the technique (see Section 2.1). Based on the obtained data, the observed effects were mainly attributed to either ionic or particulate matter. An overview of the particle number, ionic content, and particle size is provided in Table 2.

Among the averaged results of the first 12 h and after 48 h, no difference in particle number above the level of uncertainty was observed. In terms of the ionic content, no change was found for the ${\mathrm{Al}}_{2}\;O_{3}$ or the pure ${\mathrm{CeO}}_{2}$ NP exposure. A decrease in the ionic content was observed with the ionic cerium in the mixture. The mean average NP sizes in ${\mathrm{Al}}_{2}\;O_{3}$ NP-only exposure decreased over time. The other experiments did not result in a clear change in this parameter. Due to the comparable particle numbers as well as ionic contents over time, the material dissolution of the NPs did not have a significant impact. Therefore, the biological responses of the differentiated THP-1 were mainly attributed to their exposure and interactions with the different NPs. The decrease in the observed particle size was attributed to sedimentation over time, which could explain the non-significant changes in the other parameters as well.

The WST-1 results highlighted another aspect of these considerations. In addition to the application scheme and mode, the applied dose of the NPs had a crucial impact on the biological response. In this case, low-toxicity exposure concentrations were selected to provide insights into the uptake behavior and the biological response below a highly toxic concentration. This approach is reflected in the results, showing a slight decrease in cell viability with ${\mathrm{CeO}}_{2}$ NPs and no toxic effects for ${\mathrm{Al}}_{2}\;O_{3}$ NP exposure at either concentration. Similar results are observed for the other application scenarios as well. The viability was significantly higher after the exposure to ${\mathrm{CeO}}_{2}$ NPs first and to ${\mathrm{Al}}_{2}\;O_{3}$ NPs second compared to the exposure to ${\mathrm{CeO}}_{2}$ NPs only or the sequential exposure to the NPs in reversed order. This is in line with findings in the literature reporting possible reverting or even stimulating effects of ${\mathrm{CeO}}_{2}$ NPs [27]. Based on these findings, the fate of the NPs after their exposure was first evaluated with DI spICP-MS measurements. The experiments indicated no significant change in the observed particle number or ionic content. Therefore, the dissolution of the metallic NPs prior to any cellular interactions was not significant.

### 2.3. MW ICP-MS

**Figure 3:**
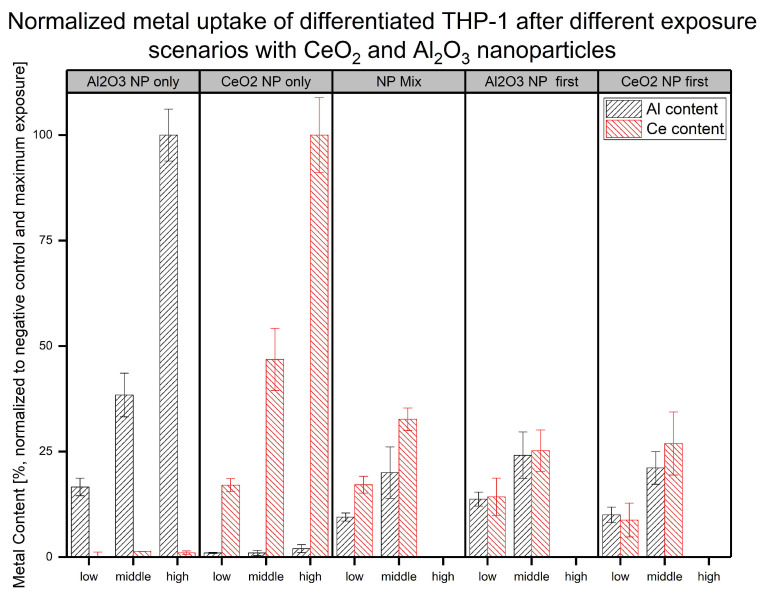
Normalized metallic contents (in untreated control and highest concentration) associated with differentiated THP-1 cells under different NP exposure scenarios (NP concentrations: low = 6 mg $L^{-1}$, middle = 12 mg $L^{-1}$, and high = 24 mg $L^{-1}$) are shown with aluminum content in black and cerium content in red from at least three biological replicates.

To determine the reason for the differences observed in the cell viability changes in the differentiated THP-1 cells after their exposure to NPs, the NP uptake according to the metallic content of the digested cells was determined. The exposed cells were digested, and the total metallic content was quantified. To further highlight changes, different exposure scenarios were used over a timeframe of 48 h. In addition to the single-exposure experiments, a stepwise exposure plan was followed. Here, the NP-containing cell culture media was changed after 24 h to the medium containing the other NPs. A more detailed overview of the experimental design can be found in Figure 1. Based on the unexposed control and the highest NP concentration used, a normalized overview of the amount of metals after cellular exposure is shown in Figure 3.

The results showed a clear difference between the determined content of the elements involved in the exposure and the observed level without additional exposure (e.g., Ce content in Al-only exposed experiment and vice versa). Furthermore, different concentrations in the same exposure scenario (single NP, mixed, and sequential exposure) led to a significant change in the metallic content. The comparison between the NP exposure experiments at the same concentration exhibited some differences in uptake. For all middle-exposure concentrations at a total NP dose of 24 µg $L^{-1}$ (12 µg $L^{-1}$ of each NP), we found similarly reduced uptake compared to the single exposure to the same element. Due to the uncertainties in the measurements, no additional insights regarding the mixed and sequential exposure were obtained for either element. For the lower total NP concentration of 12 µg $L^{-1}$ (6 µg $L^{-1}$ of each NP), more differences in uptake behavior between the exposure scenarios and metals were observed. When ${\mathrm{Al}}_{2}\;O_{3}$ NPs were applied first, the contents of both metals for experiments with sequential exposure to 12 µg $L^{-1}$ could not be distinguished from the single-NP exposure scenarios. However, in terms of the Al content, the lower concentration (6 µg $L^{-1}$ of each NP) led to significantly reduced uptake in the other co-exposure scenarios (mixed and ${\mathrm{CeO}}_{2}$ NP exposure first). This observation was the same in the sequential exposure with regard to the Ce content. Nevertheless, the mixed exposure led to a comparable measured Ce content. For Al, a significant difference in the Al levels was observed between the different scenarios. This result is in line with the difference in cell viability observed in the WST-1 assay under the different exposure scenarios. The different metallic contents implied differences in NP fates. This could have happened directly during the NP uptake stage or during the cellular response to the NP material taken up based on the exposure scenario. The results were averaged over the total cell populations, indicating broad variation and high uncertainty in most cases. Another difference between the scenarios was that the sequential exposures did not include a recovery step. Despite this, clear differences were observable, especially at a lower NP exposure concentration. However, explanations of the differences between the cellular responses to the different exposure scenarios require further investigation.

To evaluate the actual amounts of Al and Ce within the cells, MW ICP-MS experiments were performed (see Figure 3). The results showed a lower metallic content after the co-exposure experiments compared to the single-NP exposure at the higher concentration. A possible explanation might be increased toxicity due to the second exposure step (sequential scenarios) or higher total NP exposure concentration (mixed scenario). This might have led to fewer cells remaining after the last washing step. With regard to the differences between the mixed and sequential exposure experiments, no further insights were gained. In terms of the exposure to lower NP concentrations, each mixed exposure scenario produced a different uptake pattern compared to the single-NP exposure. The mixed-NP exposure resulted in significantly reduced Al content, but the Ce content was similar to that in the single-NP exposure experiment. Next, regarding the metal content, the content after the sequential exposure with ${\mathrm{Al}}_{2}\;O_{3}$ NPs first could not be distinguished from that in the single-NP exposure experiments. Finally, the sequential treatment with ${\mathrm{CeO}}_{2}$ NPs first led to a significantly reduced amount of both metals in the cells. These results further support the hypothesis of the influence of ${\mathrm{CeO}}_{2}$ NP exposure on the cellular response under the different exposure scenarios. In terms of aluminum uptake, a difference in Al levels between the two different sequential exposure scenarios at the low concentration was found. When the ${\mathrm{CeO}}_{2}$ NPs were applied first, less Al was found afterward at the lower concentration. A hypothesis for this finding is that up to a certain amount of uptaken NPs, the cells are able to effectively clear out the NPs, while, at higher concentrations, this reaction is reduced. The WST-1 results showed this behavior in terms of viability changes for the mixed exposure as well. While no adverse effects were observed at the lower exposure concentrations, higher concentrations resulted in a significant decrease in cell viability (see Figure 2 and Table 1). Below such a threshold, the induced stress might have led to different effects, such as a higher cellular activity, as indicated by the sequential exposure with ${\mathrm{CeO}}_{2}$ NPs first.

### 2.4. ToF-SIMS

**Figure 4:**
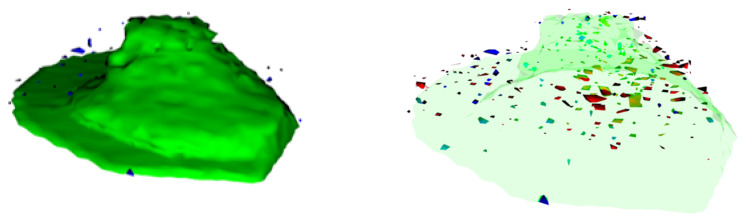
Representative ToF-SIMS 3D reconstruction of a single differentiated THP-1 cell exposed to a mix of ${\mathrm{Al}}_{2}\;O_{3}$ (blue) and ${\mathrm{CeO}}_{2}$ (red) NPs with a non-transparent (left-hand side) and a transparent (right-hand side) visualization of the signals related to the cell membrane (green). The lateral resolution obtained was roughly 80 nm, leading to a cell size of 17.2 µm and 8.6 µm.

The results of the other experiments led to further questions about the differences in the biological responses to the exposure scenarios at the single-cell level. In this study, an answer was provided by ToF-SIMS, which was the most sensitive technique applied here. Due to the high-effort and time-consuming sample preparation, this technique is best applied with a specific question in mind. The unique results can provide valuable information about the localization and impact of the entire sample within the capabilities of the instrument. Some pictures of the observed metallic contents (Al-related signals in blue and Ce-related signals in red) in and around the signals related to the cell membrane (green) are shown in Figure 4.

**Figure 5:**
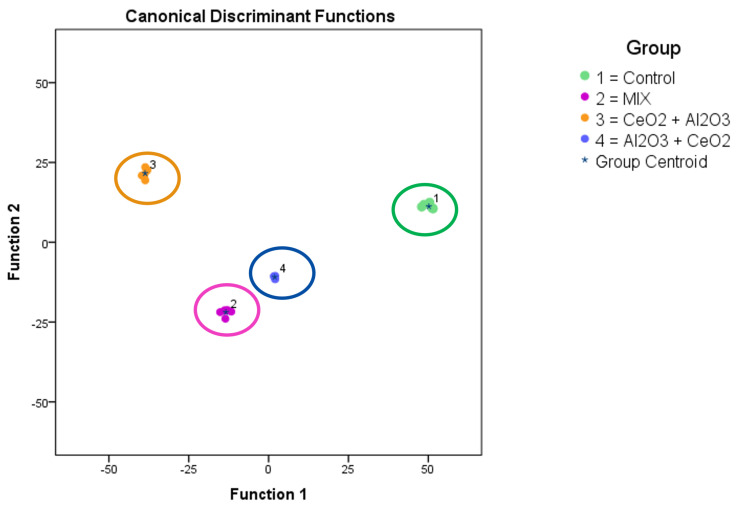
ToF-SIMS analysis of changes in the metabolited (lipids) of THP-1 cells after time-dependent treatment with ${\mathrm{CeO}}_{2}$ and ${\mathrm{Al}}_{2}\;O_{3}$. The diagram shows the values of the scores obtained from Fisher’s discriminant analysis of 6 THP−1 samples for each experiment. Group 1 shows the untreated cells (control, green). Group 2 was exposed to both particles together from time point 0 (mix, purple). Group 3 was exposed to ${\mathrm{CeO}}_{2}$ nanoparticles at time point 0 and to ${\mathrm{Al}}_{2}\;O_{3}$ nanoparticles at 24 h (${\mathrm{CeO}}_{2}$ + ${\mathrm{Al}}_{2}\;O_{3}$, orange). Group 4 was exposed to ${\mathrm{Al}}_{2}\;O_{3}$ nanoparticles at time point 0 and to ${\mathrm{CeO}}_{2}$ nanoparticles at 24 h (${\mathrm{Al}}_{2}\;O_{3}$ + ${\mathrm{CeO}}_{2}$, blue). Factor 1 in the principal component analysis accounted for 72.9% of the observed variance in the model, and factor 2 in the principal component analysis (PCA) accounted for 20.7% of the variance in the model.

The images show metallic matter in and around the cells. No larger agglomerates could be observed within the lateral resolution of the instrument. Another advantage of the ToF-SIMS measurements performed is the ability to search for systematic differences in the whole mass spectra obtained via mathematical means. These findings are summed up in Figure 5.

Based on the intensity levels of the whole spectra between 200 and 1200 *m*/*z* (see Section 3.5) a PCA was performed to distinguish significant changes in the complex datasets between the different sample treatments. A clear separation between untreated cells and the different co-exposure scenarios with the low concentration was observed. This indicated differences in the biological responses in the analyzed cells after these exposures. The observed masses, which led to significant changes in their expected molecular formula, can be used as a starting point to identify the involved mechanisms in further research.

As a next step, ToF-SIMS measurements of a subset of these samples (control cells, mixed exposure at a total of 12 µg $L^{-1}$ and both sequential exposures with 6 µg $L^{-1}$ of each NP) were performed to visualize the cellular metallic distribution and determine the systematic changes in the mass spectra. As shown with an example in Figure 4, no agglomerates were observed in any treatment. A PCA-based evaluation of the datasets from each scenario allowed for the discrimination between them. This supported the previous findings and indicated the differences in the biological responses of the differentiated THP-1 cells to the different exposure scenarios. These findings could be a starting point for identifying the interactions between NPs around or inside the cells as well as the cellular mechanisms involved. Furthermore, the impact of these interactions as well as the concentration-dependent influence on the cells, as observed at higher concentrations, could be of interest in hazard and risk assessments. The toxicity as well as other biological responses to NP exposure in increasingly complex environments are not fully understood. This includes effects of low-dose exposure, multiple-exposure schemes, recovery experiments, and different stepwise exposures to different materials, as shown here. Understanding the biological and chemical mechanisms at the cellular level could improve the safe-by-design approach of future advanced materials. This work provides a rationale for future experiments in this field.

## 3. Materials and Methods

### 3.1. Chemicals and Materials

The chemicals were purchased by Merck (Germany, Darmstadt) and had at least 98% purity, unless stated otherwise. Stock solutions of ${\mathrm{Al}}_{2}\;O_{3}$ NPs (99%, IoLiTec Ionic Liquids Technologies GmbH, Heilbronn, Germany) and ${\mathrm{CeO}}_{2}$ NPs (NM-212, Joint Research Center Ispra, Italy) were prepared in accordance with the Nanogenotox protocol: “Final protocol for producing suitable manufactured NMs exposure media” (October, 2011) [35,37]. Briefly, stock dispersions were freshly prepared by pre-wetting the NP powder with 0.5% (*v*/*v*) ethanol (96%). Afterwards, Milli-Q water (MilliPore gradient, Merck, Darmstadt, Germany) containing 0.05% BSA was added. The mixture was sonicated for 5 min and 9 s at an amplitude of 10% with a probe sonifier (200 W Bandelin Sonopuls HD 2200, BANDELIN Electronic GmbH & Co. KG, Berlin, Germany). During sonication, the sample was cooled in an ice-water bath [48]. Subsequently, the additional dilution of the NP stock solutions to 6, 12, and 24 mg $L^{-1}$ for the exposure experiments was performed. For toxicity experiments, ${\mathrm{CeO}}_{2}$ concentrations of 6 and 12 g $L^{-1}$ were used. The dissolution experiments were conducted with a concentration of 5 µg $L^{-1}$ (mixed experiment) or 50 µg $L^{-1}$ (single ${\mathrm{Al}}_{2}\;O_{3}$ NP).

### 3.2. Cell Culture

The THP-1 cell line was purchased from the German Collection of Microorganisms and Cell Cultures GmbH (DSMZ, Braunschweig, Germany). Cultivation of the cell line was performed at 37 °C with 5% ${\mathrm{CO}}_{2}$ in RPMI medium supplemented with 10% fetal calf serum, 2 mmol $L^{-1}$ L-glutamine, 10 mmol $L^{-1}$ HEPES, 1 mmol $L^{-1}$ pyruvate, 100 U ${\mathrm{mL}}^{-1}$ penicillin, and 0.1 g $L^{-1}$ streptomycin. As described in the literature, the differentiation into macrophage-like cells was achieved by adding 100 µg $L^{-1}$ phorbol-12-myristate-13 acetate [17,65]. The different exposure scenarios contained either one or two 24-h exposure steps with NP dispersions. For the two-step exposure, the cells were exposed to the NP dispersion of the other kind of NP after a washing step. For the single exposure, the cells were washed with PBS and further incubated after the first exposure step to produce the same treatment and total duration before analysis.

### 3.3. WST-1 Test

The seeding (96-well plate, density per well: $10^{4}$ cells) and differentiation of the cells was followed by their incubation with different NP dispersions (6 replicates per exposure scenario). After the final treatment, the wells were washed using PBS, and, afterwards, WST-1 reagent (Roche, Basel, Switzerland) (0.5 g $L^{-1}$) was added. The cells were incubated for 2 h at 37 °C and 5% ${\mathrm{CO}}_{2}$. The light absorption of the samples was measured on a plate reader (BioTek, Bad Friedrichshall, Germany) according to the manufacturer’s instructions. The relative cell viability was calculated as the percentage of viability of the untreated cells. The mean value and its standard error (SE) is reported as the mean of at least three independent experiments. The applied exposure concentrations were 6 (low) and 12 g $L^{-1}$ (high) for ${\mathrm{CeO}}_{2}$ and 6 (low) and 12 mg $L^{-1}$ (high) for ${\mathrm{Al}}_{2}\;O_{3}$. The mixtures used either the individual low or high concentrations.

### 3.4. Mass Spectrometry

For the calibration, standard solutions of dissolved Au, Al, and Ce (1000 mg $L^{-1}$, TraceCERT^®^, Sigma-Aldrich, Darmstadt, Germany) and 3.5% nitric acid were used. Here, a technical-grade 69% nitric acid (70% *v*/*v*) from VWR, Darmstadt, Germany, was purified in a douPur quartz sub-boiling point apparatus (MLS GmbH). Reference Au NP NIST 8012 (30 nm, Gaithersburg, MD, USA) dispersions were diluted in the media to a concentration of 50 ng $L^{-1}$ to determine the matrix-matched transport efficiencies of the DI spICP-MS setup. The experimental design of the individual measurements included frequent determination of blanks, calibrations, and transport efficiency verification, as described elsewhere [51]. In the DI spICP-MS experiments, regular measurements (n = 7) were performed over 48 h. Each experiment was performed with three independent replicates and additional media blanks. The experimental details for the DI spICP analysis of the NP solutions were adopted from a published description [10]. In brief, a quadrupole ICP-MS (Thermo Scientific XSERIES II, Thermo Fisher Scientific, Waltham, MA, USA) with a PFA ST Nebulizer, a quartz cyclonic spray chamber, as well as a 2.5 mm quartz injector (all from ESI Elemental Service and Instruments GmbH, Mainz, Germany) were used. The intensities were determined as a function of time (counts per dwell-time interval) in time-resolved analysis mode. Each run had an acquisition time of 70 s with a dwell time of 3 ms. The gas flows for the plasma, the nebulizer, and the auxiliary (all Ar) were set to 13, 0.89, and 0.7 L $\min^{-1}$, respectively. All measurements were performed using the collision cell technique to avoid polyatomic interference. The injection system was a modified microFAST MC system (ESI Elemental Service and Instruments GmbH, Mainz, Germany) containing a single loop (300 µL) and two independent syringes (5 mL and 500 µL). A flow rate of 500 µL $\min^{-1}$ was applied to ensure a transient signal. The routine tuning of this system was performed with the appropriate tune solution (ESI Elemental Service and Instruments GmbH, Mainz, Germany). The determination of the threshold between the ionic background and particle signals as well as the counting of the observed particles were performed systematically based on the corrected average intensities of the ionic background in an automated fashion. The averaged particle number per measurement was used to determine the matrix-matched transport efficiency. Similarly, the particle numbers, the particle size, and the ionic background were calculated at each time point. For these calculations, an Excel spreadsheet based on the advice from https://www.wur.nl/nl/show/spicpms-procedure-version-2.htm (accessed on 18 March 2025), was used.

For the uptake experiments, the acid-assisted microwave digestion of the exposed cells was performed as sample preparation. The washed and suspended cell dispersions were mixed with 69% ${\mathrm{HNO}}_{3}$ and 30% $H_{2}\;O_{2}$ and then processed at 200 °C and 160 bar. Afterwards, a 1:50 dilution with Milli-Q water was performed prior to the ICP-MS measurements. To ensure that no carry-over effects were present during digestion, wash digestions with 69% ${\mathrm{HNO}}_{3}$ were included after each sample. Additionally, blank digestions with Milli-Q water instead of sample solutions were used for each digestion as a control. The determination of the ionic contents of these solutions was performed with a quadrupole ICP mass spectrometer (iCAP Q, Thermo Fisher Scientific GmbH, Dreieich, Germany). It was equipped with a PFA ST Nebulizer, a 2.5 mm quartz injector, and a quartz cyclonic spray chamber (all from Thermo Fisher Scientific). The gas flows for the cool and the auxiliary gas (both Ar) were set to 14 and 0.65 L $\min^{-1}$, respectively. The sample flow rate was determined as 0.33 mL $\min^{-1}$. The elements were analyzed using the collision cell technique at 5 mL $\min^{-1}$, with a collision gas of 93% He and 7% $H_{2}$. The uptake values were normalized with the contents of the untreated cells as 0 and the maximum exposure concentration (24 mg $L^{-1}$) as 1. Experiments were performed with at least three independent replicates.

### 3.5. ToF-SIMS

A ToF-SIMS V instrument (ION-TOF GmbH, Münster, Germany) of the reflectron type, equipped with a 30 keV bismuth/manganese liquid metal ion gun (LMIG) as a primary ion source, generating nano ${\mathrm{Bi}}_{x}$^*y*+^ clusters (nanoprobe 50), a 20 keV argon gas cluster ion source, both mounted at 45° with respect to the sample surface, and an electron flood gun, was used for all ToF-SIMS measurements. The ion current was 0.5 pA at 5 kHz using a Faraday cup located on a grounded sample holder. The bunching system with a pulse of 0.7 ns led to a mass resolution that usually exceeded 9000 (full width at half-maximum) at *m*/*z* < 500 in positive-ion mode. Static SIMS conditions were obtained by controlling the primary ion dose below $10^{12}$ ions ${\mathrm{cm}}^{-2}$. A pulsed electron flood gun with 20 eV electrons was used for the charge compensation of the sample. The primary ion gun scanned a field of view of 500 µm × 500 µm applying a 512 × 512-pixel measurement raster. All depth profiles were obtained in a dual-beam mode with the same instrument. ${\mathrm{Bi}}_{3}^{+}$ was selected as the primary ion using the appropriate mass filter settings. Primary and sputter ion currents were directly determined, as described above. The scanning area for imaging analysis was 200 µm × 200 µm with 512 × 512 pixels. The sputter area for each measurement was 1000 µm × 1000 µm. Surface charging was compensated for by flooding with low-energy electrons. After the alignment of the primary ion gun, a ToF-SIMS mass spectrum was generated by summing the detected secondary positively charged ion intensities and plotting them against the mass channels. The internal calibration of the mass scale was performed using a number of known secondary ions (i.e., $C_{2}H_{5}^{+}$, $C_{3}H_{7}^{+}$, and $C_{4}H_{9}^{+}$), leading to an error in calibration for all spectra of below 5 ppm. The mass spectral data were processed using the Surface Lab 5 software (ION-TOF GmbH, Münster, Germany). Statistical analyses of the ToF-SIMS data were performed as described in detail elsewhere [23,59,66,67]. In brief, the mass spectral data were binned to 1 u. Afterwards, data processing was carried out with the SPSS+ statistical package (version 21). The mass range between 200 and 1200 mass units was used to detect the significant differences between the cells treated under different exposure scenarios. To avoid contamination of the ions by salts, system contaminants, and other medium components, ions lower than mass 200 were excluded from this study. Next, each of the acquired spectra was normalized, setting the peak sum to 100%. Finally, principal component analysis (PCA) was performed using all ions. The separation of the datasets from one another was determined with a supervised model, Fisher’s discriminant analysis. The application of the cross-validation procedure based on the “leave-one-out” cross-validation formalism to the discriminant model verified its performance. Additional details can be found in Table S2.

## 4. Conclusions

The results highlight the need to understand the biological impact of the complex interactions between different kinds of NPs in scenarios more closely resembling those in real life. This entails lower exposure concentrations over prolonged time periods or recovery phases. However, data on real exposure scenarios are still scarce and challenging to obtain [68,69]. Another point of interest is the interaction of different NPs with one another and their environment after their cellular interaction, further influencing the biological response to their exposure. Evaluating these influences and deriving specific modes of actions require a combination of analytical techniques to verify and specify the results. This work highlights the advantages of different complementary techniques in identifying the impact of sequential exposure scenarios with ${\mathrm{Al}}_{2}\;O_{3}$ and ${\mathrm{CeO}}_{2}$ NPs in this specific set up. Additionally, the experimental design and characterization of the sample materials as well as the environment play a pivotal role in understanding the fundamental processes taking place at the cellular level. In this work, a stepwise approach was used to determine if there was a biological impact of NP exposure scenarios. The observed viability changes, the determined metal contents, and the mass spectral patterns provide a rationale for investigating the reasons for the results. The results of the techniques applied highlight the influence of the exposure scheme on biological responses, especially at lower exposure concentrations. Furthermore, complementary techniques such as DLS measurements might provide valuable additional insights with regards to the media-, concentration-, and time-dependent interactions of the NPs with the cell culture media, which in turn influence the biological response.

## Figures and Tables

**Table 1:** Overview of the results of the respective two sample Student *t*-tests of the different NP exposure scenarios and concentrations (${\mathrm{CeO}}_{2}$ low: 6 g $L^{-1}$, ${\mathrm{CeO}}_{2}$ high: 12 g $L^{-1}$, ${\mathrm{Al}}_{2}\;O_{3}$ low: 6 mg $L^{-1}$, ${\mathrm{Al}}_{2}\;O_{3}$ high: 12 mg $L^{-1}$) out of 3 biological replicates.

Exposure Group	Control (p)	${\mathrm{CeO}}_{2}$ Low	${\mathrm{CeO}}_{2}$ High	${\mathrm{Al}}_{2}\;O_{3}$ Low	${\mathrm{Al}}_{2}\;O_{3}$ High	Mix Low	Mix High	${\mathrm{CeO}}_{2}$ First	${\mathrm{Al}}_{2}\;O_{3}$ First
Control (p)		y	y	n	n	n	y	y	n
${\mathrm{CeO}}_{2}$ low	y		n	y	y	n	n	y	n
${\mathrm{CeO}}_{2}$ high	y	n		y	y	y	n	y	y
${\mathrm{Al}}_{2}\;O_{3}$ low	n	y	y		n	n	y	y	n
${\mathrm{Al}}_{2}\;O_{3}$ high	n	y	y	n		n	n	y	n
Mix low	n	n	y	n	n		n	y	n
Mix high	y	n	n	y	n	n		y	n
${\mathrm{CeO}}_{2}$ first	y	y	y	y	y	y	y		y
${\mathrm{Al}}_{2}\;O_{3}$ first	n	n	y	n	n	n	n	y	

Significant (*p* < 0.05) differences between samples is indicated with a green y(es) otherwise a red n(o) is used.

**Table 2:** A summary of the results of the DI spICP-MS measurements of different NP combinations of 5 µg $L^{-1}$ (mixed experiment), 50 µg $L^{-1}$ (single ${\mathrm{Al}}_{2}\;O_{3}$ NP) in cell culture media.

	Particle Number [a.u.]		Ionic Content [ng/mL]		Mean Size [nm]	
	12 h	48 h	12 h	48 h	12 h	48 h
${\mathrm{Al}}_{2}$O_3_ ^a^	442 ± 46	502 ± 42	1.37 ± 0.12	1.24 ± 0.09	183 ± 6	166 ± 3
${\mathrm{Al}}_{2}$O_3_ ^b^	985 ± 64	932 ± 53	0.29 ± 0.04	0.26 ± 0.03	129 ± 1	128 ± 1
CeO_2_ ^a^	2958 ± 41	2887 ± 72	1.39 ± 0.14	1.25 ± 0.11	67 ± 2	63 ± 2
CeO_2_ ^b^	2531 ± 59	2423 ± 66	2.66 ± 0.20	2.20 ± 0.16	84 ± 2	80 ± 2

^a^ Single NP. ^b^ Mixture of NPs. Significant differences (above the sum of uncertainties) between samples are indicated in green.

## Data Availability

The data presented in this study are available upon reasonable request.

## Supplementary Materials

The following supporting information can be downloaded at https://www.mdpi.com/article/10.3390/molecules30071647/s1, Table S1: Overview of key parameters used for NP characterization; Table S2: ToF-SIMS PCA overview of *m*/*z* values with a high peak loading.

## Abbreviations

The following abbreviations are used in this manuscript:

NM	nanomaterial
JRC	joint research centre
MW	microwave digestion
WST-1	4-[3-(4-Iodophenyl)-2-(4-nitro-phenyl)-2H-5-tetrazolio]-1,3-benzene sulfonate
spICP-MS	single-particle inductively coupled plasma–mass spectrometry
DI	direct injection
ToF-SIMS	time-of-flight–secondary ion mass spectrometry
NP	nanoparticle
PCA	principal component analysis
SE	standard error
