# Supplementary material for: Developing a Multi-Method Approach for Understanding Cellular Uptake and Biological Response: Investigating Co-Exposure of Macrophage-like Differentiated THP-1 Cells to Al2O3 and CeO2 Nanoparticles
Source: Molecules. 2025 Apr 7;30(7):1647. doi: 10.3390/molecules30071647 (PMC11990466; doi:10.3390/molecules30071647)
Supplement: Supplementary file 1 [file molecules-30-01647-s001.zip › molecules-3502685-supplementary.pdf]

Table S1:

Table S1: Overview of key parameter of the used NMs.

	Shape (TEM)	Primary Particle Size (TEM), [nm]	Hydrodynamic Size (DLS, DMEM), [nm]	Particle Size Distribution (PDI)	Zeta Potential (DI water) [mV]
CeO2	irregular etched (not spherical)[2]	<100 (showing irregular and non-homogeneous primary particle size variation.)[2]	>100[1]	0.4-0.8[1]	33 ± 2[2]
Al2O3	Grain-like[3]	10x20-50[3]	70 ± 10 vs 230 ± 60[3]	0.53-0.11[3]	Approx. 30[4]
Comment			Medium-, concentration -and time-dependent	Both NMs are polydisperse	strongly influence of media

1-Based on Llewellyn et Al. [1]

2-Based on JRC report [2]

3-Based on Krause et Al [3]

4-Based on Murdock et Al [4]

Table S2: Peaks loading high on factor 1 ($p \geq 0.98$) or on factor 2 ($p \geq 0.95$):

Ion m/e	Lipid (tentative assignment)
947	Phosphatidyl choline C48:6
885	Phosphatidyl ethanolamine C46:2
863	Phosphatidyl choline C42:6
862	Triglyceride C52:1
847	Phosphatidyl choline C40:0
846	Ceramide C32:0
845	Phosphatidyl choline C40:1
831	Phosphatidyl ethanolamine C42:1
830	Phosphatidyl glycerine C40:2

817	Phosphatidyl choline C38:1
816	Ceramide C30:3
806	Triglyceride C48:1
760	Ceramide C30:3
742	Triglyceride C44:5
726	Ceramide C24:6
715	Phosphatidyl glyceride C32:4
690	Triglyceride C40:3
682	Ceramide C20:0
524	Lyso Phosphatidylserine C18:1
520	Lyso Phosphatidyl choline C18:3
504	Lyso Phosphatidyl ethanolamine C20:3
458	Lyso Phosphatidylcholine C12:2
397	Fatty acid C26:0
395	Fatty acid C26:1

ToF-SIMS analysis of changes in the metabolite changes (lipids) of THP1 cells after time dependent treatment with CeO_2 and Al_2O_3. The diagram shows the values of the discriminant scores obtained from Fisher's discriminant analysis of 6 THP1 samples for each experiment. Group 1 shows the untreated cells. Group 2 was exposed with both particles together from time point 0. Group 3 was exposed with CeO_2 nanoparticles at time point 0 and with Al_2O_3 nanoparticles at time point 24h. Group 4 was exposed to with Al_2O_3 nanoparticles at time point 0 and to CeO_2 nanoparticles at time point 24h. Factor 1 of the principal component analysis accounted for 72.9% of the observed variance in the model and factor 2 of the principal component analysis accounted for 20.7% of the variance in the model. For the model all 1000 peaks (m/z 200 to m/z 1200, binned to 1 mass unit) were used. The performance of the discriminant model was verified by applying the cross-validation procedure based on the "leave-one-out" cross-validation formalism (100%).

[1] - Llewellyn, S.V., Conway, G.E., Zanoni, I. *et al.* Understanding the impact of more realistic low-dose, prolonged engineered nanomaterial exposure on genotoxicity using 3D models of the human liver. *J Nanobiotechnol* **19**, 193 (2021). https://doi.org/10.1186/s12951-021-00938-w

[2] - Singh, C.; Friedrichs, S.; Ceccone, G.; Gibson, P.; Jensen, K.; Levin, M.; Goenaga, I.H.; Carlander, D.; Rasmussen, K. Cerium Dioxide, NM-211, NM-212, NM-213. Characterisation and test item preparation. Scientific analysis or review LB-NA-26649-EN-C (print),LB-NA-26649-EN-N (online), European Commission's Joint Research Centre (JRC), Luxembourg (Luxembourg), 2014. https://doi.org/10.2788/80330(print),10.2788/80203(online).

[3] - Krause, B.; Meyer, T.; Sieg, H.; Kästner, C.; Reichardt, P.; Tentschert, J.; Jungnickel, H.; Estrela-Lopis, I.; Burel, A.; Chevance, S.; et al. Characterization of aluminum, aluminum oxide and titanium dioxide nanomaterials using a combination of methods for particle surface and size analysis. RSC Adv. 2018, 8, 14377–14388. https://doi.org/10.1039/C8RA00205C.

[4] - Richard C. Murdock, Laura Braydich-Stolle, Amanda M. Schrand, John J. Schlager, Saber M. Hussain, Characterization of Nanomaterial Dispersion in Solution Prior to *In Vitro* Exposure Using Dynamic Light Scattering Technique, *Toxicological Sciences*, Volume 101, Issue 2, February 2008, Pages 239–253, https://doi.org/10.1093/toxsci/kfm240
